# Three-Year Results of Comparison Between Ring- versus Non-ring-Augmented Roux-en-Y Gastric Bypass: A Randomized Control Trial

**DOI:** 10.1007/s11695-025-08034-w

**Published:** 2025-07-17

**Authors:** Mohamed Hany, Ahmed Zidan, Mohamed Ibrahim, Aly Elbahrawy, Mohamed Mourad, Anwar Ashraf Abouelnasr, Zeyad Mohamed Hany, Bart Torensma

**Affiliations:** 1 Department of Surgery, Medical Research Institute, Alexandria University, Alexandria, Egypt; 2 King Abdullah Medical City, Makkah al Mukarramah, Saudi Arabia; 3 Department of Epidemiology, Erasmus MC, Rotterdam, Netherlands; 4 Faculty of Medicine, Alexandria University, Alexandria, Egypt

**Keywords:** Ring- and non-ring-augmented RYGB, rRYGB, nrRYGB, Weight loss surgery, Recurrent weight gain, Food tolerance, Dumping, Roux en Y gastric bypass, Gastric pouch volume, Ghrelin, Leptin, Ring-augmented procedures, Metabolic bariatric surgery

## Abstract

**Background:**

Suboptimal clinical response (SCR) after laparoscopic Roux-en-Y gastric bypass (RYGB) is often attributed to gastric pouch and gastrojejunostomy dilatation. Ring placement, which includes a ring to prevent pouch dilatation, may help reduce SCR. This study compared 3-year outcomes of ring-augmented (rRYGB) versus non-ring-augmented RYGB (nrRYGB).

**Methods:**

A single-blinded, randomized controlled trial was conducted in two specialized bariatric centers, including 120 rRYGB and 120 nrRYGB patients operated on between January 2019 and March 2020. The primary endpoint was weight loss (WL), assessed by the percentage of excess weight loss (%EWL) and total weight loss (%TWL). Secondary endpoints included gastric pouch and gastrojejunostomy volumetry, complications, quality of life (QoL), food tolerance (FT), associated medical problems, and recurrent weight gain (RWG, > 20% of TWL).

**Results:**

Ninety-two nrRYGB and 96 rRYGB patients completed the 3-year follow-up. Both groups achieved significant WL (*p* < 0.001); however, rRYGB had significantly higher %EWL and %TWL (*p* = 0.012, < 0.001) and lower RWG (*p* < 0.001). At 3 years, rRYGB had smaller gastric pouch volumes, narrower gastrojejunostomy diameters, and alimentary limb diameters (*p* < 0.001), with lower FT scores (*p* < 0.001) and incidence of dumping (*p* = 0.042). Both groups showed comparable QoL improvements and resolution of associated medical problems.

**Conclusion:**

rRYGB patients had better %EWL and %TWL and less recurrent weight gain. They maintained smaller volumes of the gastric pouches, smaller diameters of the gastrojejunostomy anastomoses and the alimentary limbs, and lower dumping after 3 years. The attribution of the ring could help patients in the long term in their postoperative period.

**Supplementary Information:**

The online version contains supplementary material available at 10.1007/s11695-025-08034-w.

## Introduction

Metabolic bariatric surgery (MBS) has proven highly effective as a treatment for morbid obesity and its related associated medical problems with strong evidence of efficacy and safety [1].

Roux-en-Y gastric bypass (RYGB) has always been one of the cornerstones of MBS. RYGB was the most common MBS worldwide before 2014, forming 45% of all MBS worldwide in 2013 [2]. Later, it became the second MBS worldwide, comprising 39.6% of all MBS in 2014 and 29.5% in 2023. However, RYGB is the most common revisional BMS, with 48.2% of revisional procedures in 2023 [3, 4].

RYGB has reported successful loss of total body mass and fat mass [5]. However, weight loss (WL) in terms of recurrent weight gain (RWG) is not uncommon with this procedure, with reported RWG rates of 20–35% [6, 7].

Several factors affect WL after MBS, including hormonal mechanisms and behavioral factors such as low physical activity and maladaptive eating behavior; however, surgical factors such as a dilated gastric pouch and dilated gastrojejunostomy are also claimed causes for SCR due to loss of restriction [6–10].

The efficacy of ring-augmented Roux-en-Y gastric bypass (rRYGB) remains debated in the literature. While some systematic reviews report favorable outcomes, with Shoar et al. (2019) demonstrating significantly greater excess weight loss in ring-augmented procedures (mean difference 5.63%; 95% CI 3.26–8.00), and Pavone et al. (2024) showing a 7.6% greater 5-year EWL with rRYGB, other studies suggest no significant advantage. Magouliotis et al. (2018) found similar outcomes between procedures at 1–2 years postoperatively, and a recent RCT (2025) reported comparable 5-year total body weight loss between rRYGB (31.8%) and nrRYGB (30.5%, *p* > 0.05). These contradictory findings underscore the need for further investigation into the long-term effectiveness of ring augmentation in RYGB procedures, which prompted our current randomized controlled trial [11–15]. Furthermore, applying a non-adjustable or adjustable ring around the gastric pouch with or without resizing the pouch and/or the gastro-jejunostomy has been reported as a revisional procedure for failed WL after RYGB with a reported drop in BMI [10].

However, no studies has assessed a wide variety of outcomes, including volumetric changes and clinical outcomes such as WL, complications, dumping, and improvements in quality of life (QoL) and associated medical problems. Therefore, this study is executed.

## Methods

### Study Design

This study was a single-blinded randomized controlled trial (RCT) that compared the outcomes of primary rRYGB vs. nrRYGB throughout 3 years of follow-up conducted in two specialized bariatric centers (Medical Research Institute and Main University Hospital, Alexandria University, Egypt. The study protocol was approved and registered by the Institutional Ethics Committee, E/C S/N. R9/2018 and conformed to the precepts of the 1975 Declaration of Helsinki. All participants signed informed consent forms. The CONSORT checklist is in Appendix 1 [16].

### Study Population

This study included patients who underwent primary laparoscopic rRYGB or nrRYGB for severe obesity between January 2019 and March 2020. Before enrolling in the trial, the patients were explained the differences in the expected benefits and possible risks of the ring and non-ring-augmented procedures.

After that, all patients were informed that the institute covered the cost of the ring placement and that some would not receive the ring after the procedure and would be kept blind until the end of the 3-year follow-up. All participants signed an informed consent form before participation.

### Inclusion Criteria

Participants aged between 18 and 60 years were included with a body mass index (BMI) of ≥ 40 kg/m^2^ or BMI ≥ 35 kg/m^2^ with associated medical problems.

### Exclusion Criteria

Smoking, previous abdominal exploration surgery, previous medical or endoscopic weight loss interventions, the presence of large hiatal hernias (HHs), and intraoperative count of small intestine were excluded.

### Study Endpoints

The primary endpoint was weight loss (WL), assessed by the percentage of total WL (%TWL), the percentage of excess WL (%EWL), and recurrent weight gain (RWG).

The secondary endpoints included volumetric changes in the gastric pouch and gastro-jejunostomy anastomosis, postoperative complications, changes in quality of life (QoL) and food tolerance (FT) [17], changes in associated medical problems and post-operative laboratory test results, including gut hormones.

### Data Collection

#### Pre-operative Workup

All participants were assessed by a multidisciplinary team (MDT), including a metabolic bariatric surgeon, dietician, endocrinologist, and psychiatrist, to ensure the participants’ fitness. All participants had routine and nutritional laboratory tests, including gut hormones, ultrasound abdominal study to exclude gallstones, and upper gastrointestinal endoscopy (UGE) for assessment of gastroesophageal reflux disease (GERD) conforming to the Los Angeles (LA) classification [18] and hiatal hernia (HH). Upward displacement of the esophagogastric junction (axial length of HH) of > 2 cm above the diaphragmatic hiatus, together with the hiatus being seen wide open all the time (Hill grade IV), was needed for diagnosis of HH in this study (19) (20). An axial length of ≥ 5 cm was considered a large HH [19].

#### Lab and Hormonal Measurements

Post-operative lab tests were done at 6 months, 1 year, 2 years, and 3 years of follow-up. The fasting levels of the hormones (ghrelin and leptin) were measured pre-, 1 and 3 years. Serum samples were allowed to clot at 18–22 ℃ for 30 min and then centrifuged at 4000 rpm for 10 min at 4 °C. All measurements were analyzed according to standardized operating procedures. Hormones were determined using ELISA (EIA-2935) (DRG International, Inc. Springfield NJ, USA).

#### Perioperative Data

Operative data include operative time, concomitant procedures, intraoperative events, and length of hospital stay (LOH).

### Surgical Techniques

The same surgical team, including two experienced surgeons and four assistants, performed rRYGB. The MiniMizer Gastric Ring® (Bariatric Solutions International, Switzerland) was used (Appendix 2).

### Post-operative Workup

All patients started prophylactic Enoxaparin against thromboembolic events 12 h before surgery and continued it 24 h after surgery for at least 3 weeks [20]. Patients were discharged with prescribed oral supplements after tolerating oral liquids.

### Complications

Post-operative complications were recorded and classified into early complications that occurred in the first 30 days, and late complications arising later on, according to the Clavien-Dindo classifications [21, 22].

### Weight Loss

WL was assessed at, year-1, year-2, and year-3 of follow-up by body mass index (BMI), percentage of total weight loss (%TWL), and percentage of excess weight loss (%EWL). For the %EWL calculation, the ideal body weight (IBW) was based on an ideal BMI of 25 kg/m^2^ (IBW = 25 × (height in m^2^) (23). recurrent weight gain. > 20% of the TWL [6].

### Quality of Life

QoL was assessed using the RAND 36-item Health Survey (RAND-36) [23].

### Food Tolerance and Dumping Score

Dumping and food tolerance (FT) were assessed by the Sigstad dumping score [24] and the FT one-page questionnaire [17], respectively, at 6 months and 3 years, giving a score varying between 1 and 27, with higher scores indicating excellent eating quality.

The Sigstad dumping score evaluated the presence of 16 different symptoms following the ingestion of sweets. When present, these symptoms had scores ranging from − 4 to + 5, and a total score of 7 or more was considered positive for dumping syndrome [25].

### Multi-detector CT (MDCT)

The anatomical features of RYGB and other MBS procedures can be reliably assessed by volumetric studies using 3-D virtual gastroscopy after expanding the gastric pouch with effervescent salts for accurate assessment of dilatations [8, 26]. Multi-detector CT (MDCT) virtual gastroscopy and 3D reconstruction was performed at 6 months and 3 years, utilizing a 64 detectors scanner (Siemens SOMATOM® Perspective, Siemens Medical Solutions, Malvern, PA), to assess the total volume of the gastric pouch and the volumes of the pouch segments above and below the ring in rRYGB, the distance between the ring and the anastomosis, the diameter of the gastro-jejunostomy and the alimentary limb, and intrathoracic migration of the gastro-esophageal junction (ITM) (Appendix 3).

### Upper Gastrointestinal Endoscopy

All patients routinely underwent postoperative upper gastrointestinal endoscopy (UGE) at 1 and 3 years following surgery. The evaluations included the same parameters assessed during the pre-operative UGE, with the addition of screening for marginal ulcers (MU). A single experienced endoscopist performed all procedures to ensure consistency. Helicobacter pylori infection was assessed using a rapid urease test conducted on endoscopic gastric biopsy specimens, and eradicated before surgery.

### Statistical Analysis

For the analyses, we used descriptive and inferential statistics. All data were first tested for normality using the Kolmogorov–Smirnov test, Q-Q plot, and Levene’s test. Categorical variables were presented as frequencies and percentages. Continuous variables were presented as means and standard deviations. Chi-squared test or Fisher exact test (when appropriate) was used to compare categorical variables between the two surgery groups. We used *t*-tests to compare continuous variables between groups.

Two analyses were conducted for the weight loss outcomes: a complete data analysis that included all patients who had at least the 1-year follow-up visit and a complete cases analysis for patients who had all 3-year follow-up visits.

Generalized estimating equation (GEE) analyses were conducted to estimate the mean differences between the two surgery groups with regards to the weight loss outcomes (weight, BMI, %TWL, and %EWL) at each follow-up year and to the RAND SF-36 at year 3. McNemar test was used to compare the changes in proportions of associated medical problems between baseline and year 3 follow-up in the complete cases’ patients. All analyses used a significance level of 0.05 and were conducted using R software version 4.2.2.

### Randomization

A single-blinded randomization procedure was performed in which patients and outpatient clinic nurses were blinded for the whole study period. The surgeon was informed of the allocation after the patient was under anesthesia. Randomized block randomization was performed using computer-generated blocks of two or four block sizes. When the patient was lost to follow-up, the therapy allocation was explained by a letter, and the patient could always contact the clinic.

### Data Capture

The analysis was performed on a blinded dataset after the medical/scientific review was completed. All protocol violations were identified and resolved, and the dataset was declared complete. All data were collected in a data management system (Castor EDC, Amsterdam, The Netherlands; https://www.castoredc.com), handled according to Good Clinical Practice guidelines, Data Protection Directive certificate, and complied with Title 21 CFR Part 11. Furthermore, the data centers where all the research data were stored were certified according to ISO27001, ISO9001, and Dutch NEN7510.

### Sample Size

The sample size calculation used the “pwr” package version 1.3–0. We considered an 8% difference in %TWL between the nrRYGB and rRYGB groups as clinically meaningful. This corresponds to a medium effect size (Cohen’s D) of 0.5 for the %TWL at any follow-up visit. Using a power of 80% with a significance level of 0.05, this resulted in a minimum sample size of 64 patients per group at any follow-up visit. Due to the expected high loss to follow-up rate over 3 years, a decision was made to include 120 patients per group [27].

## Results

### Baseline Characteristics

This single-blinded RCT included 120 patients in each of the nrRYGB and rRYGB groups (*n* = 240). The results showed no significant differences in the two groups’ baseline characteristics (Table 1).

**Table 1:** Baseline characteristics of the sample cohort

Baseline characteristics	nrRYGB (*n* = 120)	rRYGB (*n* = 120)	*p*
Age, mean ± SD	46.4 ± 6.8	45.9 ± 7.7	0.601
Sex (female), *n* (%)	97 (80.8)	101 (84.2)	0.610
**Anthropometrics**			
Height (m), mean ± SD	1.6 ± 0.1	1.6 ± 0.1	0.920
Weight (kg), mean ± SD	118.3 ± 11.3	118.1 ± 9.6	0.873
Ideal body weight (kg), mean ± SD	65.8 ± 5.5	65.8 ± 6.0	0.944
Excess weight (kg), mean ± SD	52.5 ± 9.3	52.3 ± 8.1	0.884
BMI, mean ± SD	45.0 ± 3.7	45.1 ± 3.7	0.937
**Imaging**			
Hiatal hernia, *n* (%)	27 (22.5)	25 (20.8)	0.876
Calcular cholecystitis, *n* (%)	5 (4.2)	7 (5.8)	0.769
**Endoscopy**			
Hiatal hernia, *n* (%)	27 (22.5)	25 (20.8)	0.876
LA Grade A esophagitis, *n* (%)	10 (8.33)	11 (9.17)	1.000
LA Grade B esophagitis, *n* (%)	2 (1.7)	1 (0.8)	1.000
**Associated medical problems**			
Osteoarthritis, *n* (%)	18 (15.0)	21 (17.5)	0.726
Dyslipidemia, *n* (%)	17 (14.2)	18 (15.0)	1.000
Diabetes mellitus, *n* (%)	14 (11.7)	14 (11.7)	1.000
Hypertension, *n* (%)	11 (9.2)	12 (10.0)	1.000
Sleep apnea, *n* (%)	12 (10.0)	13 (10.8)	1.000
Cardiac ischemia, *n* (%)	2 (1.7)	3 (2.5)	1.000
**RAND-36**			
Physical functioning, mean ± SD	55.1 ± 5.4	55.3 ± 5.6	0.764
Role physical, mean ± SD	56.9 ± 6.1	57.2 ± 6.4	0.721
Bodily pain, mean ± SD	69.4 ± 6.9	69.3 ± 7.0	0.927
General health perception, mean ± SD	44.1 ± 7.3	43.9 ± 7.5	0.861
Social functioning, mean ± SD	69.0 ± 8.4	69.2 ± 8.8	0.820
Role emotional, mean ± SD	56.8 ± 8.1	57.1 ± 8.7	0.741
Energy/fatigue, mean ± SD	52.8 ± 8.4	51.7 ± 9.0	0.329
Emotional, mean ± SD	60.3 ± 8.1	59.2 ± 8.6	0.312
PHC, mean ± SD	56.4 ± 6.1	56.4 ± 6.4	0.938
MHC, mean ± SD	59.7 ± 8.1	59.3 ± 8.7	0.716
Total score, mean ± SD	58.0 ± 7.0	57.9 ± 7.4	0.858

*nrRYGB* non-ring augmented Roux-en-Y gastric bypass, *rRYGB* ring augmented Roux-en-Y gastric bypass, *BMI* body mass index, *LA* Los Angeles esophagitis class, *PHC* physical health composite score, *MHC* mental health composite score. *Statistically significant (*p* < 0.05)

### Loss to Follow-Up

**Fig. 1:**
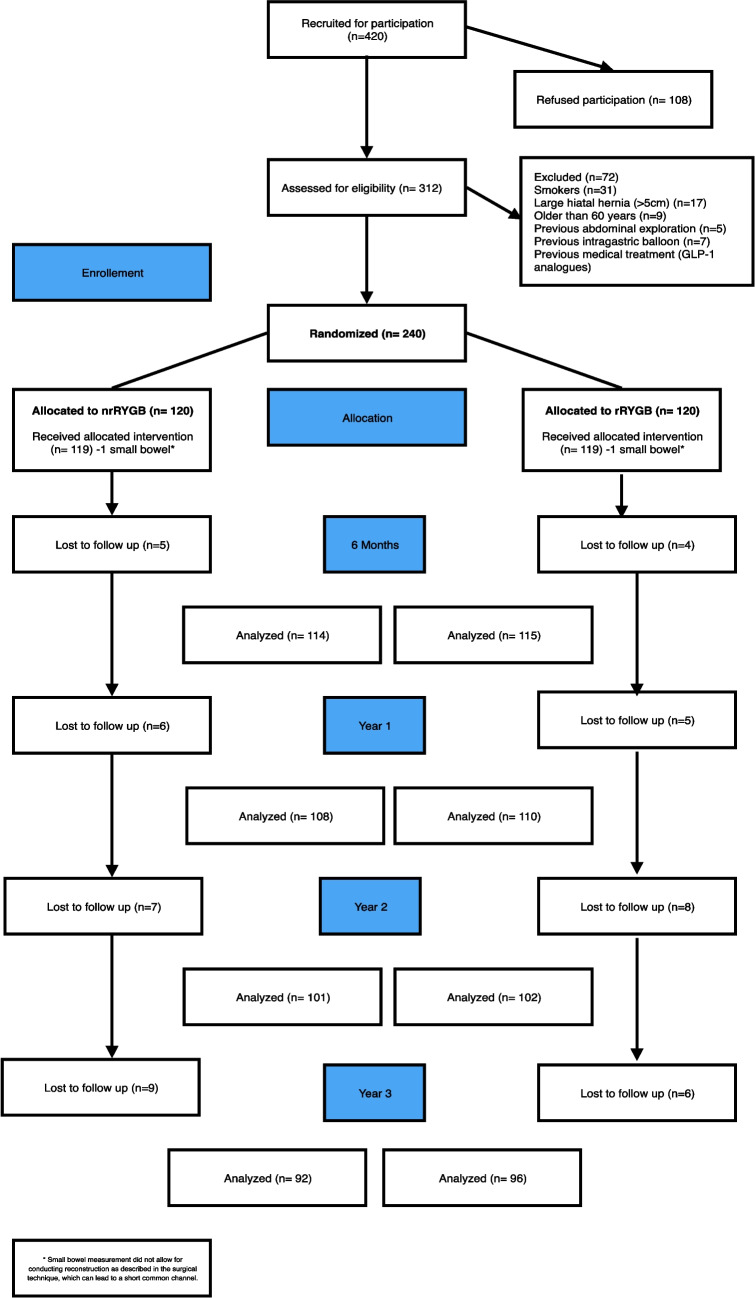
Consort flowchart

Throughout the 3-year duration, the loss to follow-up was as follows: 12 nrRYGB and 10 rRYGB patients were lost to follow-up at year 1; 19 nrRYGB and 18 rRYGB patients at year 2; and 28 nrRYGB and 24 rRYGB patients at year 3, with an overall drop rate of 21.7% at year 3 (*p* = 0.932) (CONSORT flow diagram, Fig. 1 and Appendix 4).

### Operative and Post-operative Data

**Fig. 2:**
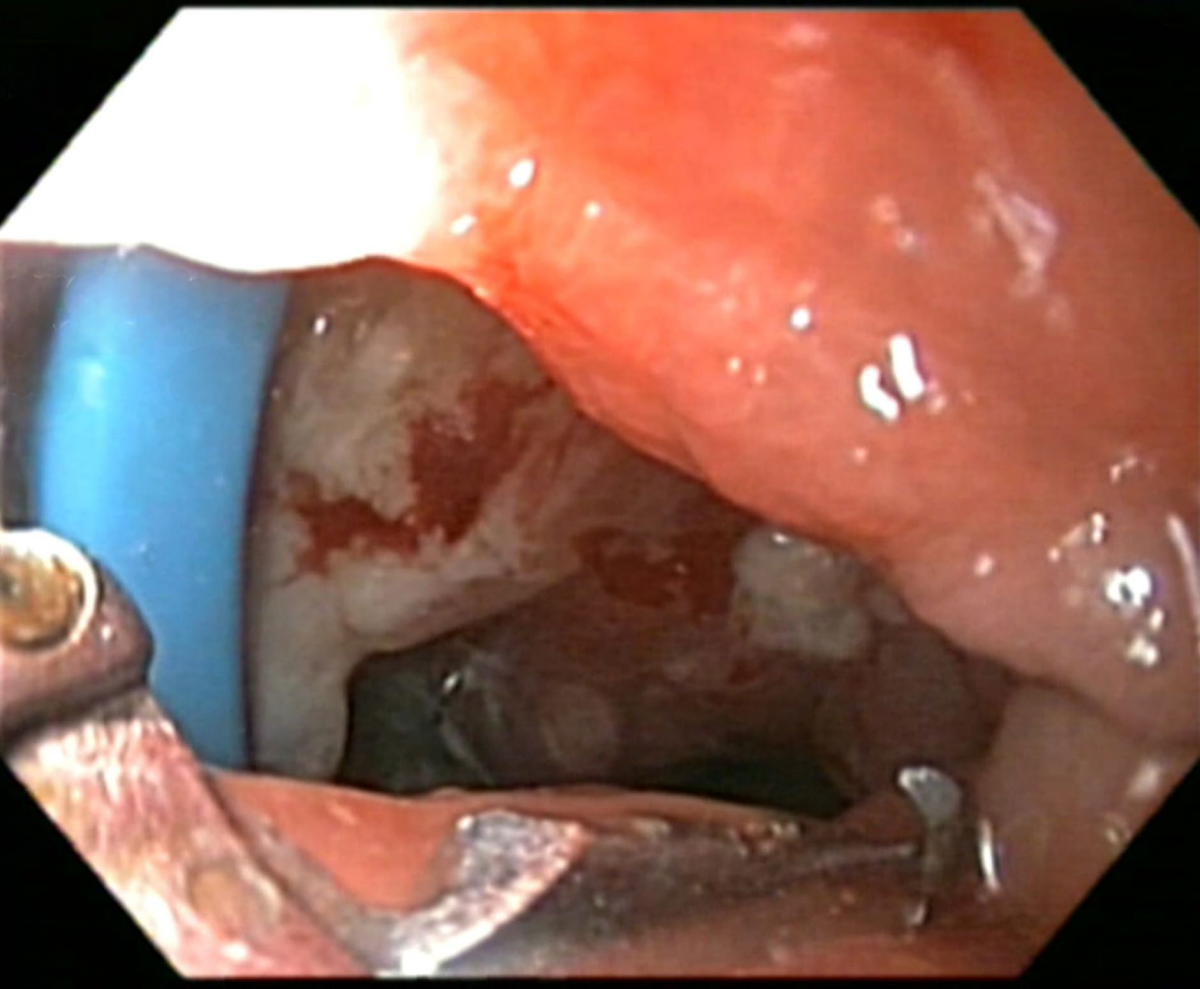
Erosion of the ring

Operation time was significantly shorter in the nrRYGB group (88.3 ± 3.1 min) compared to the rRYGB group (93.0 ± 3.0 min) (*p* < 0.001). There were no significant differences between the two groups in early (5.8% vs. 6.7% p1.000) and late complications (24.2% vs. 25.8% *p* = 0.882). One case (0.8%) in the rRYGB had erosion of the ring (Fig. 2) and was re-operated and fully recovered. Readmissions were not significantly different (8.3% vs. 9.2% *p* = 1.000), with mainly fluid resuscitation in patients with persistent vomiting and poor oral intake (8 patients) or for endoscopy in patients with melaena or persistent symptoms related to Gastroesophageal reflux disease (GERD) or marginal ulcer (MU) (7 patients) Furthermore, no significant differences in reoperations (1.7% vs. 3.3% *p* = 0.684), and endoscopy in years 1 and 3 (Table 2).

**Table 2:** Operative and postoperative data

Operative and postoperative data	nrRYGB (*n* = 120)	rRYGB (*n* = 120)	*p*
Operative time (min)	88.3 ± 3.1	93.0 ± 3.0	< 0.001*
LOH (days)	2.3 ± 0.8	2.4 ± 1.0	0.474
Extra LOH (days)	2.2 ± 0.8	2.5 ± 1.1	0.354
Combined surgery			
Cholecystectomy	4 (3.3)	5 (4.2)	1.000
Hiatal hernia repair	26 (21.6)	23 (19.2)	1.000
Cholecystectomy and hiatal hernia repair	1 (0.8)	2 (1.7)	1.000
***Overall complications***	36 (30.0)	38 (31.7)	0.889
Early complications	7 (5.8)	8 (6.7)	1.000
Hemorrhage	1 (0.8)	2 (1.7)	1.000
Melena	2 (1.7)	1 (0.8)	1.000
Vomiting	4 (3.3)	5 (4.2)	1.000
Late complications	29 (24.2)	31 (25.8)	0.882
Hiatal hernia	10 (10.8)	11 (11.5)	1.000
Marginal ulcer	4 (3.3)	3 (2.5)	1.000
Internal hernia	0 (0.0)	1 (0.8)	1.000
Port-site hernia	1 (0.8)	1 (0.8)	1.000
Anemia	3 (2.5)	5 (4.2)	0.722
Denovo GERD	2 (1.7)	2 (1.7)	1.000
Erosion	0 (0.0)	1 (0.8)	1.000
Dysphagia	0 (0.0)	1 (0.8)	1.000
Calcular cholecystitis	15 (12.5%)	14 (11.7)	1.000
**Clavien-Dindo classification**			
I	4 (3.3)	5 (4.2)	1.000
II	2 (1.7)	1 (0.8)	1.000
III-b	1 (0.8)	2 (1.7)	1.000
**Readmission**	10 (8.3)	11 (9.2)	1.000
**Reoperation**	2 (1.7)	4 (3.3)	0.684
**Reoperation cause**			
Exploration for early complications	1 (0.8)	2 (1.7)	1.000
Exploration for internal hernia	0 (0.0)	1 (0.8)	1.000
Port-site hernia repair	1 (0.8)	1 (0.8)	1.000
**Endoscopy year 1**			
Denovo hiatal hernia	2 (1.9)	1 (0.9)	0.620
Denovo LA Grade A esophagitis	2 (1.9)	1 (0.9)	0.620
Marginal ulcer	1 (0.9)	0 (0.0)	0.495
H pylori	3 (2.8)	4 (3.6)	1.000
**Endoscopy year 3**			
Denovo hiatal hernia	10 (10.8)	11 (11.5)	1.000
Denovo LA Grade A esophagitis	2 (2.2)	2 (2.1)	1.000
Marginal ulcer	4 (4.3)	3 (3.1)	0.716
H pylori	4 (4.3)	5 (5.2)	1.000

*nrRYGB* non-ring augmented Roux en-Y gastric bypass, *rRYGB* ring augmented Roux en-Y gastric bypass, *LOH* length of hospital stay, *MVO* mesenteric vascular occlusion, *DVT* deep vein thrombosis, *LA* Los Angeles esophagitis class

^*^Statistically significant (*p* < 0.05)

### Weight Loss Outcomes

No significant differences in weight, BMI, %TWL, or %EWL were observed between the two groups in year 1 and year 2 of follow-up. At year 1, the %TWL was 34.9 ± 1.4% in the nrRYGB group and 35.1 ± 1.5% in the rRYGB group (mean difference, 0.2; 95% CI, − 0.2 to 0.6; *p* = 0.347), while the %EWL was 79.5 ± 7.7% and 80.7 ± 8.5%, respectively (mean difference, 1.2; 95% CI, − 0.9 to 3.3; *p* = 0.268). At year 2, the %TWL was 40.5 ± 1.4% in the nrRYGB group and 40.7 ± 1.5% in the rRYGB group (mean difference, 0.3; 95% CI, − 0.1 to 0.7; *p* = 0.161), with %EWL of 91.9 ± 8.3% and 93.8 ± 10.2%, respectively (mean difference, 1.8; 95% CI, − 0.7 to 4.4; *p* = 0.159).

Nevertheless, in year 3, rRYGB showed statistically significant higher %TWL (40.3 ± 2.1% vs. 39.0 ± 2.5%) and %EWL (92.9 ± 11.2% vs. 89.1 ± 9.5%) compared to nrRYGB, with mean differences of 1.3 (95% CI, 0.6 to 2.0; *p* < 0.001) and 3.8 (95% CI, 0.8 to 6.7; *p* = 0.012) in %TWL and %EWL, respectively.

Additionally, the rRYGB group demonstrated a significantly lower mean RWG in kg than nrRYGB (0.8 ± 1.6 kg vs. 2.1 ± 2.4 kg), with a mean difference of − 1.3 kg (95% CI, − 0.7 to − 1.8, *p* < 0.001). Moreover, the mean percentage of RWG per maximum weight loss was significantly lower in rRYGB (1.7 ± 3.3% vs. 4.4 ± 5.0%), with a mean difference of − 2.7% (95% CI, − 1.4 to − 3.9, *p* < 0.001) (Table 3).

**Table 3:** Post-operative weight, BMI, %TWL, and %EWL after nrRYGB and rRYGB through 3 years follow-up (complete data analysis)

	nrRYGB	rRYGB	Mean difference: rRYGB–nrRYGB (95% CI)	*p*
**Year 1**	***N* = 108**	***N* = 110**		
Weight (kg)	77.1 ± 6.5	76.5 ± 5.3	-0.4 (-3.5, 2.7)	0.811
BMI	29.4 ± 2.2	29.1 ± 2.3	-0.3 (-1.4, 0.8)	0.595
%TWL	34.9 ± 1.4	35.1 ± 1.5	0.2 (-0.2, 0.6)	0.347
%EWL	79.5 ± 7.7	80.7 ± 8.5	1.2 (-0.9, 3.3)	0.268
**Year 2**	***N* = 101**	***N* = 102**		
Weight (kg)	70.5 ± 6.2	69.7 ± 5.5	-0.5 (-3.6, 2.5)	0.728
BMI	26.9 ± 2.0	26.6 ± 2.2	-0.3 (-1.4, 0.8)	0.550
%TWL	40.5 ± 1.4	40.7 ± 1.5	0.3 (-0.1, 0.7)	0.161
%EWL	91.9 ± 8.3	93.8 ± 10.2	1.8 (-0.7, 4.4)	0.159
**Year 3**	***N* = 92**	***N* = 96**		
Weight (kg)	72.0 ± 6.7	70.3 ± 6.0	-1.5 (-4.7, 1.7)	0.360
BMI	27.4 ± 2.2	26.8 ± 2.5	-0.7 (-1.8, 0.5)	0.252
%TWL	39.0 ± 2.5	40.3 ± 2.1	1.3 (0.6, 2.0)	< 0.001*
%EWL	89.1 ± 9.5	92.9 ± 11.2	3.8 (0.8, 6.7)	0.012*
Nadir weight (kg)	69.9 ± 6.3	69.4 ± 5.5	-0.5 (-1.2, 2.1)	0.599
RWG (Kg)	0.8 ± 1.6	2.1 ± 2.4	-1.3 (-0.7, -1.8)	< 0.001*
%RWG per Max weight loss (RWG/Max)	4.4 ± 5.0	1.7 ± 3.3	-2.7 (-1.4, -3.9)	< 0.001*
RWG > 20% of the TWL)	2 (2.2%)	0 (0.0%)		0.238
%EWL < 50%	0 (0.0%)	0 (0.0%)		1.000

*nrRYGB* non-ring augmented Roux en-Y gastric bypass, *rRYGB* ring augmented Roux en-Y gastric bypass, *BMI* body mass index, *%TWL* percentage of total weight loss, *%EWL* percentage of excess weight loss, *RWG* recurrent weight gain. > 20% of the TWL

^*^Statistically significant (*p* < .05)

**Fig. 3:**
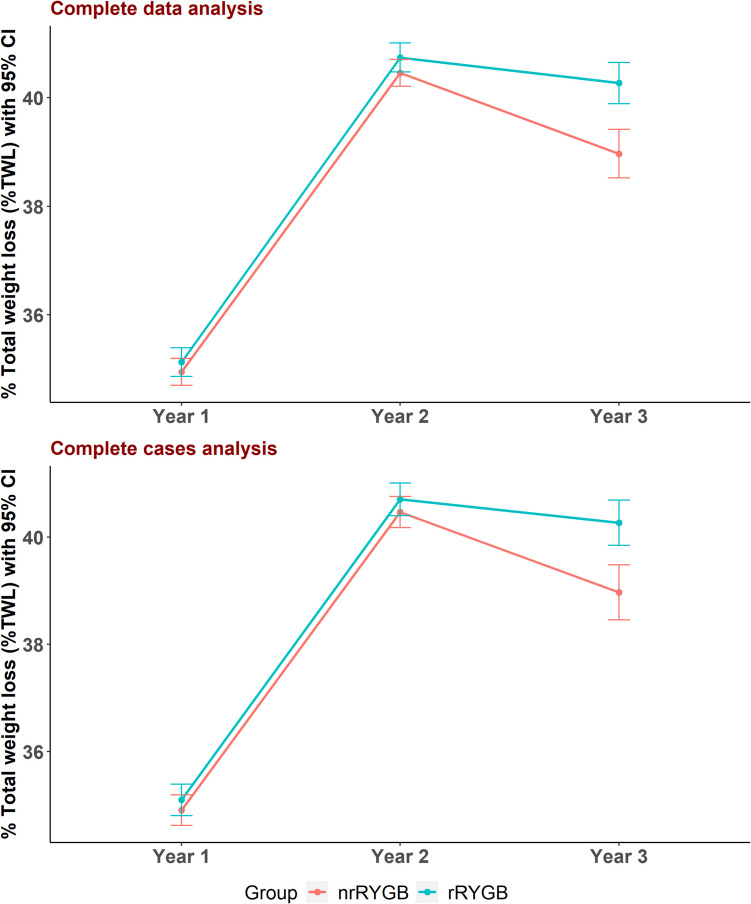
% TWL in nrRYGB and rRYGB throughout follow-up *nrRYGB*. *nrRYGB*: non-ring augmented Roux en-Y gastric bypass, *rRYGB*: ring augmented Roux en-Y gastric bypass, *%TWL*: percentage of total weight loss

**Fig. 4:**
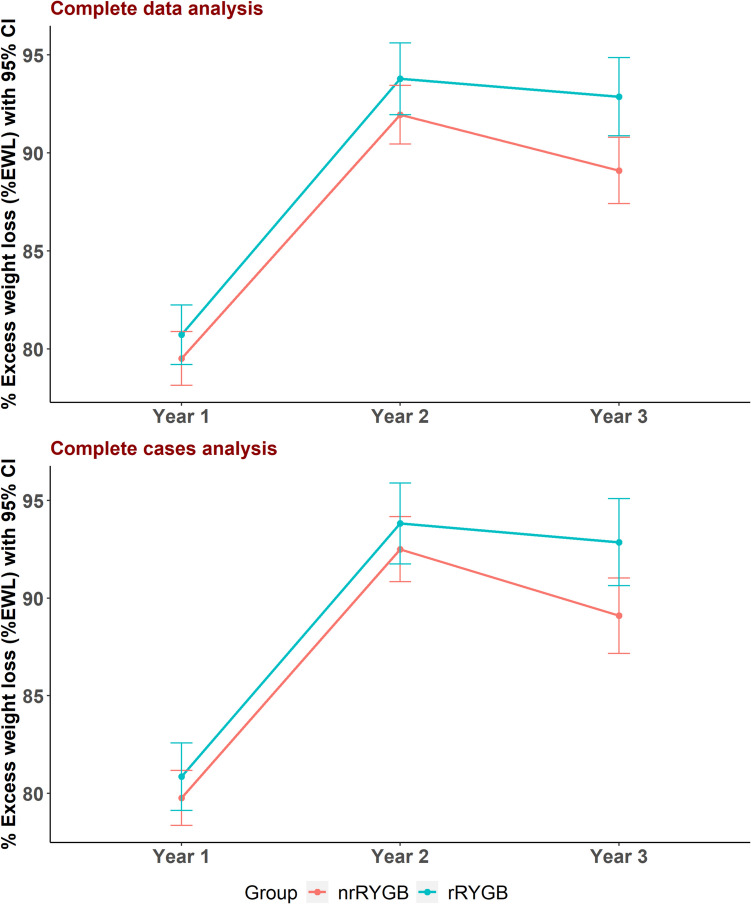
% EWL in nrRYGB and rRYGB throughout follow-up. *nrRYGB*: non-ring augmented Roux en-Y gastric bypass, *rRYGB*: ring augmented Roux en-Y gastric bypass, *%EWL*: percentage of excess weight loss

After adjusting for confounding factors (rRYGB vs. nrRYGB), we found that both groups significantly changed %TWL and %EWL across the follow-up years (*p* < 0.001). Moreover, rRYGB had higher overall mean differences in %TWL and %EWL compared to nrRYGB, with a mean difference throughout the study period of 0.4 (95% CI, 0.2, 0.6, *p* < 0.001) in %TWL and 1.6 (95% CI, 0.5, 2.6, *p* = 0.003) in %EWL (Figs. 3, 4 and Table 4). The analysis of common channel lengths immediately after surgery confirmed baseline equivalence between the nrRYGB and rRYGB groups. The mean common channel length was 437.3 ± 49.3 cm in the nrRYGB group and 430.2 ± 44.0 cm in the rRYGB group, with no significant difference (*p* = 0.241). This equivalence remained consistent across all analyzed cohorts, despite patient dropout, with no significant differences observed at any follow-up period (6 months, 1 year, 2 years, and 3 years) (Appendix 6).

**Table 4:** GEE analysis for the mean difference in % TWL and % EWL over time relative to year 1 adjusted for the surgery type (main effects) and common channel lengths

Term	% TWL		% EWL	
	Mean difference (95% CI)	*p*	Mean difference (95% CI)	*p*
Year 2 vs. year 1	5.6 (5.3, 5.8)	< 0.001*	12.7 (11.1, 14.4)	< 0.001*
Year 3 vs. year 1	4.6 (4.2, 5.0)	< 0.001*	10.9 (9.1, 12.7)	< 0.001*
rRYGB vs. nrRYGB	0.4 (0.2, 0.6)	< 0.001*	1.6 (0.5, 2.6)	0.003*

*nrRYGB* non-ring augmented Roux en-Y gastric bypass, *rRYGB* ring augmented Roux en-Y gastric bypass, *%TWL* percentage of total weight loss, *%EWL* percentage of excess weight loss

^*^Statistically significant (*p* < .05)

### Quality of Life

There were no statistically significant differences in the RAND-36 score outcomes between the nrRYGB and rRYGB groups in post-operative year 3 (Appendix 7). When adjustments for surgery type, there were statistically significant improvements in all domains and total scores after surgery compared to pre-baseline (*p* < 0.001), with no significant differences detected between both procedures (Appendix 8).

### Associated Medical Problems

The incidences of associated medical problems throughout follow-up in the study groups showed no significant differences between both groups regarding the resolution of associated medical problems. Furthermore, both groups showed significant resolution in associated medical problems at post-operative year 3 compared to baseline (Appendix 9).

### Postoperative Laboratory Assessment

Laboratory investigations at 6 months, 1 year, and 2 years revealed significant differences in hemoglobin levels across all time points, consistently lower in the rRYGB group compared to the nrRYGB group. Specifically, hemoglobin levels were 11.9 ± 1.6 g/dL vs. 12.4 ± 1.5 g/dL at 6 months (*p* = 0.021), 11.7 ± 1.6 g/dL vs. 12.3 ± 1.5 g/dL at 1 year (*p* = 0.022), and 11.5 ± 1.6 g/dL vs. 12.1 ± 1.5 g/dL at 2 years (*p* = 0.022).

At the 1-year follow-up, the rRYGB group also observed significant elevations in leptin (15.2 ± 1.4 ng/mL vs. 14.7 ± 1.5 ng/mL, *p* = 0.021) and ghrelin (244.2 ± 42.3 pg/mL vs. 226.6 ± 42.4 pg/mL, *p* = 0.005) levels. No significant differences were detected in other laboratory parameters at any time point (Appendix 5).

### Volumetry

There were no significant differences between both groups at 6-months of follow-up; nevertheless, at post-operative year 3, the mean total gastric pouch volume, mean gastrojejunostomy diameter, and mean alimentary limb diameter were significantly higher in the nrRYGB than rRYGB (*p* < 0.001). The incidences of intra-thoracic migration of the gastric pouch (ITM) were not significantly different in both groups.

**Fig. 5:**
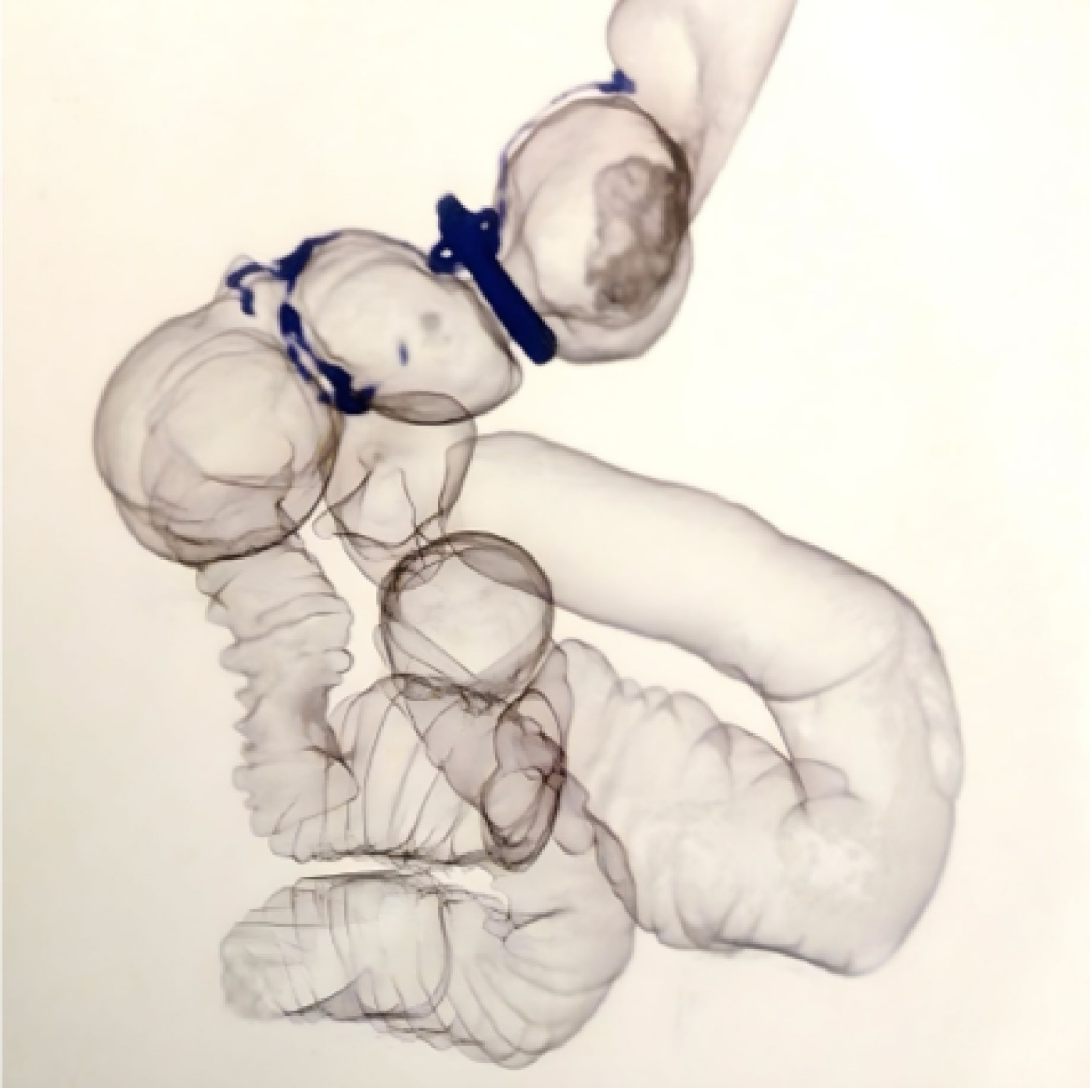
Volumetric scan

The volumetric measurements of the gastric pouch in the rRYGB group demonstrated significant changes between 6 months and 3 years postoperatively. The mean volume of the pouch segment above the ring increased significantly from 25.7 ± 3.9 mL at 6 months to 41.9 ± 5.5 mL at 3 years (*p* < 0.001). In contrast, the mean volume of the pouch segment below the ring decreased significantly from 16.9 ± 3.1 mL at 6 months to 14.3 ± 3.3 mL at 3 years (*p* < 0.001). Additionally, the mean distance between the ring and the anastomosis was significantly reduced, from 2.4 ± 0.5 cm at 6 months to 1.5 ± 0.5 cm at 3 years (*p* < 0.001) (Fig. 5, Table 5).

**Table 5:** Pouch volumetry at 6 months and 3 years postoperatively in the RYGB and BRYGB grous and change in the pouch volumetry items specific to the BRYGB group at 6 months and 3 years postoperatively assessed in the complete cases (*n* = 96)

Volumes	nrRYGB	rRYGB	*p*
**6 months**	***N* = 114**	***N* = 115**	
Total pouch volume (ml)	42.4 ± 5.6	41.8 ± 5.9	0.431
Anastomosis size (cm)	2.1 ± 0.5	2.0 ± 0.4	0.170
ITM, n (%)	13 (10.8%)	14 (11.7%)	1.000
Migration distance of ITM (cm)	1.1 ± 0.3	1.0 ± 0.2	0.907
Diameter of alimentary limb (cm)	2.0 ± 0.4	1.9 ± 0.5	0.057
**3 years**	***N* = 92**	***N* = 96**	
Total pouch volume (ml)	72.4 ± 6.1	55.7 ± 6.9	< 0.001*
Anastomosis size (cm)	3.2 ± 0.5	1.8 ± 0.5	< 0.001*
ITM, n (%)	37 (30.8%)	45 (37.5%)	0.341
Migration distance of ITM (cm)	1.2 ± 0.5	1.2 ± 0.5	0.522
Diameter of alimentary limb (cm)	3.3 ± 0.6	2.1 ± 0.5	< 0.001*
**Volumes**	**6 months**	**3 years**	***p***
Volume of the pouch above the ring (ml)	25.7 ± 3.9	41.9 ± 5.5	< 0.001*
Volume of the pouch below the ring (ml)	16.9 ± 3.1	14.3 ± 3.3	< 0.001*
Distance between the band and the anastomosis (cm)	2.4 ± 0.5	1.5 ± 0.5	< 0.001*

*nrRYGB* non-ring augmented Roux en-Y gastric bypass, *rRYGB* ring augmented Roux en-Y gastric bypass, *ITM* intra-thoracic migration of the gastric pouch. *Statistically significant (*p* < 0.05)

### Food Tolerance and Dumping

At 6 months of follow-up, no significant differences were observed between the nrRYGB and rRYGB groups in the mean FT score or the incidence of dumping, as determined by a Sigstad score ≥ 7.

However, by the 3-year follow-up, the nrRYGB group demonstrated significantly higher mean FT scores (*p* < 0.001) and a significantly greater incidence of dumping on the Sigstad score (*p* = 0.042) compared to the rRYGB group (Table 6).

**Table 6:** Dumping scores and food tolerance (FT) at 6 months and 3 years of follow-up

	nrRYGB	rRYGB	*p*
**At 6-months of follow-up**	***N* = 114**	***N* = 115**	
Patients with Sigstad score ≥ 7, n (%)	88 (77.2%)	82 (71.3%)	0.386
FT score at 6 months, mean ± SD	21.7 ± 1.5	21.2 ± 2.4	0.062
**At 3-years of follow-up**	***N* = 92**	***N* = 96**	
Patients with Sigstad score ≥ 7, n (%)	51 (55.4)	38 (39.6)	0.042*
FT score at 3 years, mean ± SD	24.0 ± 1.4	22.5 ± 2.5	< 0.001*

*nrRYGB* non-ring augmented Roux en-Y gastric bypass, *rRYGB* ring augmented Roux en-Y gastric bypass *Statistically significant (*p* < 0.05)

## Discussion

This was a single-blinded RCT conducted in two specialized bariatric centers that included 120 patients in each of the nrRYGB and rRYGB groups (*n* = 240) over a 3-year follow-up period.

### Weight Loss Effects

Both groups significantly changed %TWL and %EWL across the follow-up years. However, these changes were significantly higher in the rRYGB. Furthermore, rRYGB had significantly higher %TWL and %EWL at year 3 of follow-up. Also, rRYGB had a significantly lower mean RWG in kg, and a lower mean percentage of RWG per maximum weight loss.

A 2018 meta-analysis by Magouliotis et al. that analyzed the data of 2249 rRYGB patients and 1650 nrRYGB patients reported significantly better %EWL at 5 years with rRYGB with %EWL ranging between 61.6% and 74.0% compared to %EWL ranging from 59.8% to 65.2% in nrRYGB. However, there were no significant differences throughout the first 5 years after surgery [13]. The ring-augmented pouch maintains restriction with less dilation of the pouch and the gastrojejunal anastomosis over time, which might play a role in maintaining weight loss over time after RYGB [9, 13].

A 2014 meta-analysis by Buchwald et al. analyzed the data of 8707 rRYGB patients from 15 observational studies, and reported a %EWL of 72.5% at 5 years and 69.4% at 10 years after rRYGB [12].

Another meta-analysis of RCTs by Shoar et al. in 2018, which included 494 patients from 3 RCTs with follow-ups of 2 and 5 years, reported 5% greater EWL with rRYGB, with %EWL ranging from 42 to 92% compared to %EWL ranging from 44 to 89% in the nrRYGB [11].

A recent meta-analysis by Pavone et al. in 2024, analyzed the data of 3230 rRYGB and 5302 nrRYGB patients from 13 studies and showed a 6.03% significant increase in %EWL in rRYGB at post-operative year-1, a 5.32% greater %EWL in rRYGB at year-2, however non-significant, and a 7.6% greater %EWL in rRYGB at year-5 [14].

The findings of this study confirm the WL benefits of rRYGB over non-ring augmented procedures at intermediate follow-up, although initial WL outcomes were comparable. It is important to note that while statistically significant, these differences were relatively small and only emerged at the 3-year time point, raising questions about their clinical significance and implications for routine practice.

### Volumetric Measurements

The volumetric assessment in this study showed no significant differences between rRYGB and nrRYGB at 6 months of follow-up, and by the time, the nrRYGB group experienced significantly more dilated gastric pouches, gastrojejunostomy dimeters and alimentary limb diameters at 3 years. This data confirms the role of the ring in maintaining restriction and preventing dilatation which is believed to be one of the mechanisms of maintaining better WL in rRYGB. Moreover, the pouch proximal to the ring has shown significant dilatation over time, but this dilatation did not compromise the overall pouch size which remained significantly lower than nrRYGB.

A study by Robert et al. correlated the RYGB volumes at 3 and 12 months of follow-up using 3D virtual gastroscopy to WL and reported no correlation between WL and RYGB volumes at 1 year of follow-up [8]. This could be explained by the short follow-up period of 1 year, which does not allow for significant dilatation. However, data from the studies assessing the effect of resizing the gastric pouch and the gastro-jejunostomy for RWG after RYGB show improvement in weight loss, which indicates the effect dilatation after RYGB as a mechanism for SCR that can be fixed by restoration of restriction. Similarly, a study by Ferro et al. 2022 reported significant WL and decreased BMI at 2 years after resizing the dilated gastric pouch and/or gastro-jejunostomy for RWG after RYGB and one anastomosis gastric bypass (OAGB) [28].

### Complications

In this study, there were no significant differences in the incidence of early and late complications between the study groups. When comparing this with data from several meta-analyses, it also showed no significant differences in overall complication rates between rRYGB and nrRYGB [11, 13, 14, 29].

The incidence of MU in this study was 3.3% in nrRYGB and 2.5% in rRYGB at 3 years of follow-up as diagnosed by routine EGD, with no significant differences. Both rRYGB and nrRYGB have comparable incidences of MU in the literature [11–13].

Nevertheless, this was between rRYGB and nrRYGB. For a critical question about pouch length and marginal ulcer formation in context from an epidemiological perspective, the relationship between pouch length and MU formation presents an interesting case study in time-dependent associations and statistical power. Our studies align with short- and intermediate-term data from comparable studies. Boerboom et al., in their RCT of extended versus standard pouches (*n* = 132), found only two cases in the standard pouch group and one case in the extended pouch group (*p* = 0.662) [30]. Similarly, Safari et al. reported zero cases in both extended and standard pouch groups (*n* = 219) at 12 months [31]. However, longer-term data suggests a potential time-dependent effect. Gao et al. demonstrated that after 6 years, MU incidence was significantly higher in patients with large pouches (23.7%, 9/38) compared to those with standard pouches (6.4%, 3/47, *p* = 0.023) [32].

This time-dependent variation raises important methodological considerations. Our study, like many others in this field, may be underpowered to detect differences in relatively rare events like MU, especially if the effect emerges gradually. Using epidemiological principles, we must consider whether the time frame of observation is sufficient to capture the full development of the outcome of interest.

Additionally, multiple confounding factors beyond pouch length influence MU formation, including surgical technique, smoking status, NSAID use, and Helicobacter pylori infection. Our standardized protocol controlled for these variables, which strengthens internal validity but may limit the detection of pouch-specific effects if they are relatively modest. Longer follow-up with our cohort will be essential to determine whether the temporal patterns observed in other studies emerge in our population. This highlights the methodological importance of extended observation periods when studying outcomes with potentially delayed manifestation.

Denovo LA Grade A esophagitis was diagnosed in two patients in each group in this study at year 3 of follow-up by visualizing esophageal mucosal breaks during routine UGE. One of them was asymptomatic, while three patients had symptoms of heartburn. All patients diagnosed with pre-operative GERD had complete resolution of GERD by UGE. Accordingly, the ring application did not affect the resolution of GERD or development of de-novo GERD in this study. These data coincide with the literature as rRYGB and nrRYGB have reported similar rates of GERD remissions [13].

HH is standard among MBS candidates, with high reported rates in the literature reaching 30–50% [33]. The incidences of HH in this study were 22.5% and 20.8% in nrRYGB and RYGB groups, respectively, as diagnosed by routine pre-operative UGE, considering that patients with large HH were excluded from the study.

### Ring Erosion

In this study, we had one case of ring erosion that presented at 30 months after rRYGB with persistent abdominal pain, vomiting, intolerability to oral feeding, and melaena. Ring erosion is a rare but well-known complication specific to ring-augmented MBS procedures. rRYGB has reported rates of ring erosion ranging from 0% to 7.7%. Other ring-related complications include ring slippage and herniation of the small bowel with small bowel obstruction [12, 13].

### Food Tolerance and Dumping Syndrome

In this study, the mean FT score was almost equal in both groups at 6 months of follow-up. However, at 3 years, the mean FT score was significantly lower in the rRYGB group, indicating less tolerance to food intake in the rRYGB, which might have a role in the maintained better weight loss outcomes. This coincides with data from the literature showing more frequent vomiting and less food tolerance, especially to meat and bread, with rRYGB [11, 13, 34, 35].

Dumping syndrome is a common problem after metabolic bariatric surgery, particularly after RYGB, with reported rates of up to 70% in the intermediate follow-up [36, 37]. Using the validated Sigstad scoring system, we found interesting temporal patterns in dumping syndrome presentation. Initially, at 6 months post-surgery, no significant differences in dumping syndrome rates were observed between groups. However, by 3 years of follow-up, the nrRYGB group had a significantly higher incidence of dumping (55.4%) compared to the rRYGB group (39.6%). The ring augmentation may promote more consistent food passage and regulate gastric emptying over time, potentially reducing dumping frequency despite causing greater food intolerance. These findings highlight the complex relationship between surgical technique, food tolerance, and dumping physiology in the years following RYGB procedures.

### Postoperative Laboratory Assessment

An interesting finding was that mean leptin and ghrelin levels were significantly higher in the rRYGB group at 3 years compared to nrRYGB. This change in hormones might be a part of the mechanism of maintained weight loss in the rRYGB in this study, besides other possible factors such as maintained restriction and less food tolerance but needs to be studied more.

### Associated Medical Problems

No significant differences were recorded between the study groups regarding the resolution of associated medical problems throughout follow-up. Equal resolution of associated medical problems has been reported in the literature following both rRYGB and nrRYGB [13, 29, 34, 35, 38].

## Limitations

While this study was rigorously designed with randomized groups and robust statistical techniques, certain limitations should be acknowledged. Although follow-up was comprehensive, it is currently limited to 3 years, which is insufficient to draw definitive conclusions. Longer-term data on weight loss, late complications, and deficiencies would provide a more complete picture of the interventions’ effects. Future studies could expand on these findings by using other types of MBS and extended follow-up durations.

## Conclusion

rRYGB demonstrated superior %EWL and %TWL outcomes, along with reduced weight recurrence, compared to nrRYGB. The clinical relevance of this small but statistically significant difference remains unknown, and longer-term follow-up is needed. These advantages were accompanied by smaller gastric pouch volumes, narrower gastrojejunostomy anastomoses and alimentary limbs, and a lower incidence of dumping syndrome at 3 years postoperatively, including a ring potential benefits the enhancement of long-term outcomes by maintaining the restrictive and functional integrity of the surgical alterations. These findings underscore the potential benefits of ring-augmented procedures in Roux-en-Y gastric bypass and highlight the need for continuous research into their application. Long-term multicenter studies assessing outcomes such as weight maintenance, quality of life, nutritional deficiencies, and metabolic improvements will be essential to refine these techniques and optimize patient care.

## Supplementary Information

Below is the link to the electronic supplementary material.

Supplementary file1 (DOCX 28 KB)

Supplementary file2 (DOCX 17 KB)

Supplementary file3 (DOCX 14 KB)

Supplementary file4 (DOCX 20 KB)

Supplementary file5 (DOCX 25 KB)

Supplementary file6 (DOCX 15 KB)

Supplementary file7 (DOCX 16 KB)

Supplementary file8 (DOCX 16 KB)

Supplementary file9 (DOCX 15 KB)

## Data Availability

Data is provided within the manuscript or supplementary information files.
