# Supplementary material for: Three-Year Results of Comparison Between Ring- versus Non-ring-Augmented Roux-en-Y Gastric Bypass: A Randomized Control Trial
Source: Obes Surg. 2025 Jul 17;35(8):2812–27. doi: 10.1007/s11695-025-08034-w (PMC12380956; doi:10.1007/s11695-025-08034-w)
Supplement: Supplementary file 2 — Supplementary file2 (DOCX 17 KB) [file 11695_2025_8034_MOESM2_ESM.docx]

**Appendix II: Surgical techniques**

***Appendix II-A: The nrRYGB Operation***

Standard 5 ports were used three 12-mm ports (for the camera, right and left working ports) and two 5-mm ports (for liver retraction and for the assistant). Pneumo-peritoneum was created after using optical trocars for entry. Starting with dissection of the lesser omentum around 4 cm above the crow’s foot to create a window to pass the stapler at about 10 cm below the angle of His to start creating the gastric pouch, followed by vertical stapling over a 40 fr bougie towards the angle of His with dissection of the angle of His and all posterior gastric adhesions. Blue reloads were used for creation of the gastric pouch. After counting the whole bowel length, a length of 100cm for each of the alimentary limb and the biliopancreatic limb was attempted in all cases while always keeping a common limb length of at least 300cm. blue reloads were used for construction of the gastro-jejunostomy, and jejuno-jejunal anastomoses. The stapling defects were closed with continuous sutures using 3/0 V-Loc 180 sutures (Covidien, Mansfield, MA, USA). The gastric pouch was measured by tape to ensure at least 8 cm length above the gastro-jejunostomy. The staple lines were reinforced with invaginating series using the same barbed sutures. Closure of mesenteric defects was attempted in all cases using 3/0 V-Loc non-absorbable sutures. Crural repair for hiatal hernia using 2/0 V-Loc non-absorbable sutures was attempted in all cases with pre-operatively diagnosed hiatal hernia. Concomitant cholecystectomy was performed in in all cases with pre-operatively diagnosed calcular cholecystitis. Intra-operative methylene blue leak test was routinely performed. A tube drain was routinely placed in the left sub-phrenic space.

**Key features of nrRYGB procedure**

| **Bougie size** | 40 fr |
| --- | --- |
| **Width of pouch** | 2-2.5 cm |
| **First stapler fire (Lower pouch limit)** | Above the level of incisura angularis (10cm below angle of His) |
| **Last stapler fire** | 1–1.5 cm lateral to esophago-gastric junction |
| **His angle dissection** | Yes |
| **Length of pouch** | 8-10 cm above the gastro-jejunostomy |
| **Capacity of pouch** | 35-40 ml |
| **Counting the whole bowel length** | yes |
| **Limb lengths** | Alimentary limb 100cm  Biliopancreatic limb 100cm  Always keeping a common limb length of at least 300cm. |
| **Width of gastroenterostomy** | 2-2.5 cm |
| **Reinforcement** | Oversewing invaginating sero-muscular sutures |
| **Hiatal hernia repair** | Yes, if pre-operatively diagnosed |
| **Methylene blue test** | yes |

***Appendix II-B: The rRYGB Operation***

Same standard 5 ports were used. The creation of gastric pouch and the anastomoses were similar to nrRYGB, followed by perigastric dissection to insert the MiniMizer Gastric Ring® (Bariatric Solutions International, Switzerland) at about 3 cm above the gastro-jejunostomy. The ring was loosely placed around the pouch and adjusted to a size that allows the passage of a 5 mm instrument through the ring beside the pouch, mostly a size of 7.5 cm (1.75 cm internal diameter) and fixed in place by two non-absorbable sutures passing through the built-in holes in the ring. Concomitant procedures, methylene blue leak test, were also performed similar to nrRYGB. A tube drain was routinely placed.

**Key features of rRYGB procedure**

| **Bougie size** | 40 fr |
| --- | --- |
| **Width of pouch** | 2-2.5 cm |
| **First stapler fire (Lower pouch limit)** | Above the level of incisura angularis (10cm below angle of His) |
| **Last stapler fire** | 1–1.5 cm lateral to esophago-gastric junction |
| **His angle dissection** | Yes |
| **Length of pouch** | 8-10 cm above the gastro-jejunostomy |
| **Capacity of pouch** | 35-40 ml |
| **Position of the ring** | 3 cm above the gastro-jejunostomy |
| **Counting the whole bowel length** | yes |
| **Limb lengths** | Alimentary limb 100cm  Biliopancreatic limb 100cm  Always keeping a common limb length of at least 300cm. |
| **Width of gastroenterostomy** | 2-2.5 cm |
| **Reinforcement** | Oversewing invaginating sero-muscular sutures |
| **Hiatal hernia repair** | Yes, if pre-operatively diagnosed |
| **Methylene blue test** | yes |
