# Supplementary material for: Three-Year Results of Comparison Between Ring- versus Non-ring-Augmented Roux-en-Y Gastric Bypass: A Randomized Control Trial
Source: Obes Surg. 2025 Jul 17;35(8):2812–27. doi: 10.1007/s11695-025-08034-w (PMC12380956; doi:10.1007/s11695-025-08034-w)
Supplement: Supplementary file 3 — Supplementary file3 (DOCX 14 KB) [file 11695_2025_8034_MOESM3_ESM.docx]

**Appendix 3: MDCT**

Patients were instructed to fast for a minimum of four hours prior to the examination. An intravenous injection of 40 mg butyl-scopolamine was administered, after which patients were asked to ingest two to four packs of effervescent granules (sodium bicarbonate) as tolerated, without water, while positioned on the examination table. Image acquisition was conducted in the supine position, targeting only the stomach, which was adequately inflated with gas as observed on the tomogram. Scans were obtained using a low-radiation protocol with the following parameters: 80 kV, 125 mA, 32 × 0.6 mm collimation, and a reconstructed slice thickness of 1 mm, employing SAFIRE iterative reconstruction techniques. The imaging data were subsequently transferred to a dedicated three-dimensional (3D) workstation for analysis. Volume-rendered 3D images were generated through a combination of manual and semi-automatic segmentation methods, with distinct masks created to represent various anatomical structures in different colors. The entire procedure, including interpretation, was performed by a single radiologist for all patients.
