# Supplementary material for: Three-Year Results of Comparison Between Ring- versus Non-ring-Augmented Roux-en-Y Gastric Bypass: A Randomized Control Trial
Source: Obes Surg. 2025 Jul 17;35(8):2812–27. doi: 10.1007/s11695-025-08034-w (PMC12380956; doi:10.1007/s11695-025-08034-w)
Supplement: Supplementary file 4 — Supplementary file4 (DOCX 20 KB) [file 11695_2025_8034_MOESM4_ESM.docx]

**Appendix 4: Baseline characteristics of the complete cases analysis**

| **Baseline characteristics** | nrRYGB  (n = 92) | rRYGB  (n = 96) | p |
| --- | --- | --- | --- |
| Age | 46.8 ± 6.7 | 45.7 ± 7.4 | 0.601 |
| Sex (female), n (%) | 73 (79.3) | 80 (83.3) | 0.610 |
| **Anthropometrics** |  |  |  |
| Height (m), mean±SD | 1.6 ± 0.1 | 1.6 ± 0.1 | 0.920 |
| Weight (kg), mean±SD | 118.1 ± 11.4 | 117.7 ± 9.5 | 0.873 |
| Ideal body weight (kg), mean±SD | 65.8 ± 5.5 | 65.9 ± 5.9 | 0.944 |
| Excess weight (kg), mean±SD | 52.3 ± 8.7 | 51.8 ± 8.2 | 0.884 |
| BMI, mean±SD | 44.9 ± 3.3 | 44.8 ± 3.7 | 0.937 |
| **Imaging** |  |  |  |
| Hiatal hernia, n (%) | 20 (21.7) | 21 (21.9) | 0.876 |
| Calcular cholecystitis, n (%) | 5 (5.4) | 7 (7.3) | 0.769 |
| **Endoscopy** |  |  |  |
| Hiatal hernia, n (%) | 20 (21.7) | 21 (21.9) | 0.876 |
| GERD grade A, n (%) | 7 (7.6) | 11 (11.5) | 0.517 |
| GERD grade B, n (%) | 2 (2.2) | 1 (1.0) | 1.000 |
| **Associated medical problems** |  |  |  |
| Osteoarthritis, n (%) | 16 (17.4) | 16 (16.7) | 0.726 |
| Dyslipidemia, n (%) | 15 (16.3) | 16 (16.7) | 1.000 |
| Diabetes mellitus, n (%) | 11 (12.0) | 14 (14.6) | 1.000 |
| Hypertension, n (%) | 10 (10.9) | 10 (10.4) | 1.000 |
| Sleep apnea, n (%) | 8 (8.7) | 12 (12.5) | 1.000 |
| Cardiac ischemia, n (%) | 2 (2.2) | 3 (3.1) | 1.000 |
| **Lab investigations** |  |  |  |
| Hemoglobin (g/dl), mean±SD | 12.6 ± 1.5 | 12.9 ± 1.6 | 0.261 |
| Ferritin (ug/l), mean±SD | 121.3 ± 13.9 | 126.0 ± 13.8 | 0.319 |
| WBC, mean±SD | 5.8 ± 1.0 | 6.0 ± 0.9 | 0.112 |
| SGOT, mean±SD | 28.4 ± 4.8 | 28.4 ± 5.1 | 0.972 |
| SGPT, mean±SD | 39.2 ± 4.6 | 39.0 ± 5.1 | 0.749 |
| Urea, mean±SD | 27.4 ± 7.1 | 25.9 ± 6.7 | 0.359 |
| Creatinine, mean±SD | 0.8 ± 0.2 | 0.8 ± 0.2 | 0.757 |
| INR, mean±SD | 1.0 ± 0.0 | 1.0 ± 0.0 | 0.577 |
| fT3 (pmol/l), mean±SD | 4.0 ± 0.7 | 3.9 ± 0.7 | 0.548 |
| fT4 (pmol/l), mean±SD | 22.4 ± 4.1 | 21.6 ± 4.0 | 0.523 |
| TSH (mIU/l), mean±SD | 2.4 ± 0.9 | 2.2 ± 0.9 | 0.312 |
| Fasting glucose (mg/dl), mean±SD | 90.8 ± 12.4 | 91.8 ± 14.7 | 0.737 |
| HbA1c (%), mean±SD, mean±SD | 5.1 ± 0.9 | 5.2 ± 1.0 | 0.799 |
| Cholesterol (mg/dl), mean±SD | 171.7 ± 37.6 | 174.7 ± 34.1 | 0.382 |
| TG (mg/dl), mean±SD | 140.2 ± 24.6 | 141.5 ± 26.2 | 0.711 |
| LDL (mg/dl), mean±SD | 86.7 ± 21.1 | 88.5 ± 20.3 | 0.444 |
| Albumin (gm/dl), mean±SD | 4.1 ± 0.4 | 4.0 ± 0.4 | 0.850 |
| Ca (mg/dl), mean±SD | 9.5 ± 0.5 | 9.5 ± 0.5 | 0.803 |
| Vit D (ng/ml), mean±SD | 33.2 ± 7.3 | 34.0 ± 7.5 | 0.705 |
| B12 (pg/ml), mean±SD | 573.6 ± 120.9 | 587.3 ± 129.0 | 0.903 |
| PTH (pg/ml), mean±SD | 37.7 ± 8.3 | 38.3 ± 8.1 | 0.836 |
| HOMA-IR, mean±SD | 1.8 ± 0.5 | 1.7 ± 0.5 | 0.925 |
| INSULIN, fasting (mU/l), mean±SD | 8.0 ± 2.2 | 8.0 ± 2.4 | 0.793 |
| PYY, fasting (pg/ ml), mean±SD | 98.8 ± 13.3 | 99.3 ± 13.8 | 0.744 |
| GLP1, fasting (pmol/l), mean±SD | 7.2 ± 0.5 | 7.2 ± 0.5 | 0.754 |
| GIP, fasting (pg/ml), mean±SD | 68.3 ± 5.8 | 68.4 ± 5.7 | 0.845 |
| Leptin, fasting (ng/ml), mean±SD | 30.6 ± 1.4 | 30.5 ± 1.4 | 0.581 |
| Ghrelin, fasting (pg/ml), mean±SD | 324.9 ± 41.1 | 325.8 ± 41.3 | 0.241 |
| **RAND SF-36** |  |  |  |
| Physical functioning, mean±SD | 55.1 ± 5.5 | 55.1 ± 5.5 | 0.764 |
| Role physical, mean±SD | 56.9 ± 6.1 | 57.0 ± 6.3 | 0.721 |
| Bodily pain, mean±SD | 69.1 ± 6.8 | 69.2 ± 6.9 | 0.927 |
| General health perception, mean±SD | 43.8 ± 7.3 | 43.8 ± 7.3 | 0.861 |
| Social functioning, mean±SD | 68.7 ± 8.4 | 69.0 ± 8.6 | 0.820 |
| Role emotional, mean±SD | 56.6 ± 8.2 | 57.0 ± 8.6 | 0.741 |
| Energy/fatigue, mean±SD | 52.7 ± 8.5 | 51.6 ± 8.9 | 0.329 |
| Emotional, mean±SD | 60.1 ± 8.1 | 59.0 ± 8.6 | 0.312 |
| PHC, mean±SD | 56.2 ± 6.2 | 56.3 ± 6.3 | 0.938 |
| MHC, mean±SD | 59.6 ± 8.2 | 59.2 ± 8.6 | 0.716 |
| Total score, mean±SD | 57.9 ± 7.0 | 57.7 ± 7.3 | 0.858 |

***nrRYGB:*** *Non-ring augmented roux en-Y gastric bypass,* ***rRYGB:*** *ring augmented roux en-Y* ***gastric*** *bypass* ***BMI***: body mass index, ***GERD***: gastro-esophageal reflux disease*,* ***PHC:*** physical health composite score***, MHC:*** mental health composite score. *Statistically significant (p < 0.05)
