# Supplementary material for: Three-Year Results of Comparison Between Ring- versus Non-ring-Augmented Roux-en-Y Gastric Bypass: A Randomized Control Trial
Source: Obes Surg. 2025 Jul 17;35(8):2812–27. doi: 10.1007/s11695-025-08034-w (PMC12380956; doi:10.1007/s11695-025-08034-w)
Supplement: Supplementary file 5 — Supplementary file5 (DOCX 25 KB) [file 11695_2025_8034_MOESM5_ESM.docx]

**Appendix 5: postoperative Lab investigations**

|  |  |  |  |
| --- | --- | --- | --- |
| **Lab investigations at 6 months** | nrRYGB  (n = 114) | rRYGB  (n = 115) | p |
| Hemoglobin (g/dl), mean±SD | 12.4 ± 1.5 | 11.9 ± 1.6 | 0.021* |
| Ferritin (ug/l), mean±SD | 113.1 ± 17.4 | 111.6 ± 14.6 | 0.530 |
| WBC, mean±SD | 5.2 ± 1.0 | 5.3 ± 0.9 | 0.330 |
| SGOT, mean±SD | 34.6 ± 5.3 | 33.4 ± 5.4 | 0.114 |
| SGPT, mean±SD | 32.6 ± 5.2 | 32.0 ± 6.3 | 0.513 |
| Urea, mean±SD | 26.4 ± 7.2 | 26.7 ± 6.5 | 0.767 |
| Creatinine, mean±SD | 1.3 ± 0.3 | 1.3 ± 0.3 | 0.636 |
| INR, mean±SD | 1.5 ± 0.1 | 1.5 ± 0.1 | 0.988 |
| fT3 (pmol/l), mean±SD | 5.0 ± 0.7 | 4.9 ± 0.7 | 0.809 |
| fT4 (pmol/l), mean±SD | 25.6 ± 4.2 | 24.9 ± 4.0 | 0.192 |
| TSH (mIU/l), mean±SD | 2.4 ± 1.0 | 2.4 ± 0.9 | 0.913 |
| Fasting glucose (mg/dl), mean±SD | 82.6 ± 6.1 | 82.8 ± 6.1 | 0.824 |
| HbA1c (%), mean±SD, mean±SD | 5.2 ± 0.6 | 5.3 ± 0.6 | 0.689 |
| Cholesterol (mg/dl), mean±SD | 126.4 ± 25.0 | 125.5 ± 15.2 | 0.757 |
| TG (mg/dl), mean±SD | 107.2 ± 15.4 | 110.4 ± 16.5 | 0.170 |
| LDL (mg/dl), mean±SD | 58.8 ± 10.3 | 61.4 ± 10.7 | 0.095 |
| Albumin (gm/dl), mean±SD | 4.2 ± 0.4 | 4.2 ± 0.4 | 0.615 |
| Ca (mg/dl), mean±SD | 9.4 ± 0.5 | 9.4 ± 0.5 | 0.879 |
| Vit D (ng/ml), mean±SD | 28.4 ± 7.4 | 28.2 ± 8.1 | 0.855 |
| B12 (pg/ml), mean±SD | 596.7 ± 120.3 | 610.9 ± 128.1 | 0.437 |
| PTH (pg/ml), mean±SD | 42.5 ± 8.6 | 44.0 ± 8.6 | 0.224 |
| HOMA-IR, mean±SD | 1.5 ± 0.3 | 1.5 ± 0.3 | 0.408 |
| INSULIN, fasting (mU/l), mean±SD | 5.5 ± 1.3 | 5.2 ± 1.4 | 0.174 |
| PYY, fasting (pg/ ml), mean±SD | 179.6 ± 24.2 | 177.2 ± 23.1 | 0.495 |
| GLP1, fasting (pmol/l), mean±SD | 10.4 ± 0.5 | 10.4 ± 0.5 | 0.752 |
| GIP, fasting (pg/ml), mean±SD | 83.1 ± 6.0 | 82.2 ± 5.8 | 0.282 |

*nrRYGB: Non-ringed* roux en-Y gastric bypass, *rRYGB: ringed* roux en-Y gastric bypass, *Statistically significant (p < 0.05)

|  |  |  |  |
| --- | --- | --- | --- |
| **Lab investigations at 1 year** | nrRYGB  (n = 108) | rRYGB  (n = 110) | p |
| Hemoglobin (g/dl), mean±SD | 12.3 ± 1.5 | 11.7 ± 1.6 | 0.022* |
| Ferritin (ug/l), mean±SD | 113.0 ± 17.4 | 111.5 ± 14.6 | 0.531 |
| WBC, mean±SD | 5.1 ± 1.0 | 5.2 ± 0.9 | 0.323 |
| SGOT, mean±SD | 34.5 ± 5.3 | 33.3 ± 5.4 | 0.112 |
| SGPT, mean±SD | 32.5 ± 5.2 | 31.9 ± 6.3 | 0.522 |
| Urea, mean±SD | 26.3 ± 7.2 | 26.6 ± 6.5 | 0.769 |
| Creatinine, mean±SD | 1.2 ± 0.3 | 1.2 ± 0.3 | 0.598 |
| INR, mean±SD | 1.4 ± 0.1 | 1.4 ± 0.1 | 0.495 |
| fT3 (pmol/l), mean±SD | 4.9 ± 0.7 | 4.8 ± 0.7 | 0.832 |
| fT4 (pmol/l), mean±SD | 25.5 ± 4.2 | 24.8 ± 4.0 | 0.192 |
| TSH (mIU/l), mean±SD | 2.3 ± 1.0 | 2.3 ± 0.9 | 0.919 |
| Fasting glucose (mg/dl), mean±SD | 82.5 ± 6.1 | 82.7 ± 6.1 | 0.817 |
| HbA1c (%), mean±SD, mean±SD | 5.1 ± 0.6 | 5.2 ± 0.6 | 0.715 |
| Cholesterol (mg/dl), mean±SD | 126.3 ± 25.0 | 125.4 ± 15.2 | 0.756 |
| TG (mg/dl), mean±SD | 107.1 ± 15.4 | 110.3 ± 16.5 | 0.170 |
| LDL (mg/dl), mean±SD | 58.7 ± 10.3 | 61.3 ± 10.7 | 0.096 |
| Albumin (gm/dl), mean±SD | 4.1 ± 0.4 | 4.1 ± 0.4 | 0.603 |
| Ca (mg/dl), mean±SD | 9.3 ± 0.5 | 9.3 ± 0.5 | 0.943 |
| Vit D (ng/ml), mean±SD | 28.3 ± 7.4 | 28.1 ± 8.1 | 0.858 |
| B12 (pg/ml), mean±SD | 596.6 ± 120.3 | 610.7 ± 128.1 | 0.437 |
| PTH (pg/ml), mean±SD | 42.4 ± 8.6 | 43.9 ± 8.6 | 0.227 |
| HOMA-IR, mean±SD | 1.4 ± 0.3 | 1.4 ± 0.3 | 0.317 |
| INSULIN, fasting (mU/l), mean±SD | 5.4 ± 1.3 | 5.1 ± 1.4 | 0.175 |
| PYY, fasting (pg/ ml), mean±SD | 179.4 ± 24.2 | 177.1 ± 23.2 | 0.494 |
| GLP1, fasting (pmol/l), mean±SD | 10.3 ± 0.5 | 10.3 ± 0.5 | 0.782 |
| GIP, fasting (pg/ml), mean±SD | 83.0 ± 6.0 | 82.1 ± 5.8 | 0.282 |
| Leptin, fasting (ng/ml), mean±SD | 14.7 ± 1.5 | 15.2 ± 1.4 | 0.021* |
| Ghrelin, fasting (pg/ml), mean±SD | 226.6 ± 42.4 | 244.2 ± 42.3 | 0.005* |

|  |  |  |  |
| --- | --- | --- | --- |
| **Lab investigations at 2 years** | nrRYGB  (n = 101) | rRYGB  (n = 102) | p |
| Hemoglobin (g/dl), mean±SD | 12.1 ± 1.5 | 11.5 ± 1.6 | 0.022* |
| Ferritin (ug/l), mean±SD | 112.8 ± 17.4 | 111.3 ± 14.6 | 0.532 |
| WBC, mean±SD | 4.9 ± 1.0 | 5.0 ± 0.9 | 0.362 |
| SGOT, mean±SD | 34.3 ± 5.3 | 33.0 ± 5.4 | 0.110 |
| SGPT, mean±SD | 32.3 ± 5.2 | 31.7 ± 6.3 | 0.522 |
| Urea, mean±SD | 26.1 ± 7.2 | 26.4 ± 6.5 | 0.765 |
| Creatinine, mean±SD | 1.0 ± 0.3 | 1.0 ± 0.3 | 0.583 |
| INR, mean±SD | 1.2 ± 0.1 | 1.2 ± 0.1 | 0.348 |
| fT3 (pmol/l), mean±SD | 4.7 ± 0.7 | 4.6 ± 0.7 | 0.857 |
| fT4 (pmol/l), mean±SD | 25.3 ± 4.2 | 24.6 ± 4.0 | 0.191 |
| TSH (mIU/l), mean±SD | 2.1 ± 1.0 | 2.1 ± 0.9 | 0.913 |
| Fasting glucose (mg/dl), mean±SD | 82.3 ± 6.1 | 82.5 ± 6.1 | 0.820 |
| HbA1c (%), mean±SD, mean±SD | 4.9 ± 0.6 | 5.0 ± 0.6 | 0.750 |
| Cholesterol (mg/dl), mean±SD | 126.1 ± 25.0 | 125.2 ± 15.2 | 0.757 |
| TG (mg/dl), mean±SD | 106.9 ± 15.4 | 110.1 ± 16.5 | 0.171 |
| LDL (mg/dl), mean±SD | 58.5 ± 10.3 | 61.1 ± 10.7 | 0.096 |
| Albumin (gm/dl), mean±SD | 3.9 ± 0.4 | 3.9 ± 0.4 | 0.590 |
| Ca (mg/dl), mean±SD | 9.1 ± 0.5 | 9.1 ± 0.5 | 0.859 |
| Vit D (ng/ml), mean±SD | 28.1 ± 7.4 | 27.9 ± 8.1 | 0.857 |
| B12 (pg/ml), mean±SD | 596.4 ± 120.4 | 610.6 ± 128.1 | 0.437 |
| PTH (pg/ml), mean±SD | 42.2 ± 8.6 | 43.7 ± 8.6 | 0.226 |
| HOMA-IR, mean±SD | 1.2 ± 0.3 | 1.2 ± 0.3 | 0.373 |
| INSULIN, fasting (mU/l), mean±SD | 5.2 ± 1.3 | 4.9 ± 1.4 | 0.176 |
| PYY, fasting (pg/ ml), mean±SD | 179.3 ± 24.2 | 176.9 ± 23.2 | 0.494 |
| GLP1, fasting (pmol/l), mean±SD | 10.1 ± 0.5 | 10.1 ± 0.5 | 0.782 |
| GIP, fasting (pg/ml), mean±SD | 82.8 ± 6.0 | 81.9 ± 5.8 | 0.280 |

| **Lab investigations at 3 years** | nrRYGB  N = 92 | rRYGB  N = 96 | p |
| --- | --- | --- | --- |
| Hemoglobin (g/dl), mean±SD | 11.9 ± 1.5 | 11.3 ± 1.6 | 0.021* |
| Ferritin (ug/l), mean±SD | 112.6 ± 17.4 | 111.1 ± 14.6 | 0.531 |
| WBC, mean±SD | 4.7 ± 1.0 | 4.8 ± 0.9 | 0.344 |
| SGOT, mean±SD | 34.1 ± 5.3 | 32.8 ± 5.4 | 0.110 |
| SGPT, mean±SD | 32.1 ± 5.2 | 31.5 ± 6.3 | 0.519 |
| Urea, mean±SD | 25.9 ± 7.2 | 26.2 ± 6.5 | 0.769 |
| Creatinine, mean±SD | 0.8 ± 0.3 | 0.8 ± 0.3 | 0.648 |
| INR, mean±SD | 1.0 ± 0.1 | 1.0 ± 0.1 | 0.671 |
| fT3 (pmol/l), mean±SD | 4.5 ± 0.7 | 4.4 ± 0.7 | 0.842 |
| fT4 (pmol/l), mean±SD | 25.1 ± 4.2 | 24.4 ± 4.0 | 0.193 |
| TSH (mIU/l), mean±SD | 1.9 ± 1.0 | 1.9 ± 0.9 | 0.912 |
| Fasting glucose (mg/dl), mean±SD | 82.1 ± 6.1 | 82.3 ± 6.1 | 0.818 |
| HbA1c (%), mean±SD, mean±SD | 4.73 ± 0.6 | 4.76 ± 0.63 | 0.7232 |
| Cholesterol (mg/dl), mean±SD | 125.9 ± 25.0 | 125.0 ± 15.2 | 0.756 |
| TG (mg/dl), mean±SD | 106.7 ± 15.4 | 109.9 ± 16.5 | 0.170 |
| LDL (mg/dl), mean±SD | 58.3 ± 10.3 | 60.9 ± 10.7 | 0.096 |
| Albumin (gm/dl), mean±SD | 3.7 ± 0.4 | 3.7 ± 0.4 | 0.567 |
| Ca (mg/dl), mean±SD | 8.9 ± 0.5 | 8.9 ± 0.5 | 0.888 |
| Vit D (ng/ml), mean±SD | 27.9 ± 7.4 | 27.7 ± 8.1 | 0.858 |
| Vit B12 (pg/ml), mean±SD | 596.2 ± 120.3 | 610.3 ± 128.1 | 0.437 |
| PTH (pg/ml), mean±SD | 42.0 ± 8.6 | 43.5 ± 8.6 | 0.223 |
| HOMA-IR, mean±SD | 1.0 ± 0.3 | 1.0 ± 0.3 | 0.342 |
| INSULIN, fasting (mU/l), mean±SD | 5.0 ± 1.3 | 4.7 ± 1.4 | 0.175 |
| PYY, fasting (pg/ ml), mean±SD | 179.0 ± 24.2 | 176.7 ± 23.2 | 0.495 |
| GLP1, fasting (pmol/l), mean±SD | 9.9 ± 0.5 | 9.9 ± 0.5 | 0.773 |
| GIP, fasting (pg/ml), mean±SD | 82.6 ± 6.0 | 81.7 ± 5.8 | 0.282 |
| Leptin, fasting (ng/ml), mean±SD | 14.3 ± 1.5 | 14.8 ± 1.4 | 0.020* |
| Ghrelin, fasting (pg/ml), mean±SD | 228.4 ± 42.5 | 243.8 ± 42.3 | 0.014* |

***nrRYGB:*** *Non-ring augmented roux en-Y gastric bypass,* ***rRYGB:*** *ring augmented roux en-Y* ***gastric*** *bypass* *Statistically significant (p < .05)
