# Supplementary material for: Three-Year Results of Comparison Between Ring- versus Non-ring-Augmented Roux-en-Y Gastric Bypass: A Randomized Control Trial
Source: Obes Surg. 2025 Jul 17;35(8):2812–27. doi: 10.1007/s11695-025-08034-w (PMC12380956; doi:10.1007/s11695-025-08034-w)
Supplement: Supplementary file 6 — Supplementary file6 (DOCX 15 KB) [file 11695_2025_8034_MOESM6_ESM.docx]

**Appendix 6: Common Channel length**

| **Study sample** | **nrRYGB** | **rRYGB** | ***p*** |
| --- | --- | --- | --- |
| **All patients** | **N = 120** | **N = 120** |  |
| Peri operative Common channel length (cm) | 437.3 ± 49.3 | 430.2 ± 44.0 | 0.241 |
| **Patients present at 6 months** | **N = 114** | **N = 115** |  |
| Peri operative Common channel length (cm) | 443.3 ± 10.3 | 426 ± 23.0 | 0.176 |
| **Patients present at 1 year** | **N = 108** | **N = 110** |  |
| Peri operative Common channel length (cm) | 430 ± 24.5 | 425 ± 69.2 | 0.853 |
| **Patients present at 2 years** | **N = 101** | **N = 102** |  |
| Peri operative Common channel length (cm) | 473.3 ± 58.3 | 416.7 ± 64.7 | 0.115 |
| **Patients present at 3 years** | **N = 92** | **N = 96** |  |
| Peri operative Common channel length (cm) | 431.3 ± 49.0 | 432.4 ± 41.7 | 0.857 |
