# Supplementary material for: Three-Year Results of Comparison Between Ring- versus Non-ring-Augmented Roux-en-Y Gastric Bypass: A Randomized Control Trial
Source: Obes Surg. 2025 Jul 17;35(8):2812–27. doi: 10.1007/s11695-025-08034-w (PMC12380956; doi:10.1007/s11695-025-08034-w)
Supplement: Supplementary file 7 — Supplementary file7 (DOCX 16 KB) [file 11695_2025_8034_MOESM7_ESM.docx]

**Appendix 7: RAND-36 scores at year-3 after nrRYGB and rRYGB**

| **Domain** | **nrRYGB**  **(n = 92)** | **rRYGB**  **(n = 96)** | **Mean difference**  **(95% CI)** | ***p*** |
| --- | --- | --- | --- | --- |
| Physical functioning | 90.1 ± 5.8 | 91.1 ± 6.1 | 0.7 (-1.5, 2.9) | 0.524 |
| Role limitations physical | 86.1 ± 6.9 | 87.3 ± 6.9 | 0.9 (-1.6, 3.5) | 0.478 |
| Bodily pain | 80.9 ± 7.0 | 82.1 ± 7.2 | 1.3 (-1.3, 4.0) | 0.326 |
| General Health perception | 66.2 ± 7.4 | 67.0 ± 7.4 | 1.0 (-1.8, 3.9) | 0.472 |
| Social functioning | 88.6 ± 6.6 | 89.6 ± 6.0 | 0.7 (-2.2, 3.5) | 0.641 |
| Role limitations emotional | 86.7 ± 7.6 | 87.3 ± 7.6 | 0.3 (-2.8, 3.3) | 0.850 |
| Energy/fatigue | 70.1 ± 8.2 | 70.9 ± 8.8 | 1.9 (-1.4, 5.2) | 0.249 |
| Emotional | 80.7 ± 8.4 | 81.6 ± 8.8 | 1.9 (-1.3, 5.2) | 0.248 |
| PHC | 80.8 ± 6.3 | 81.9 ± 6.5 | 1.0 (-1.4, 3.4) | 0.417 |
| MHC | 81.5 ± 7.4 | 82.3 ± 7.5 | 1.2 (-1.8, 4.2) | 0.435 |
| Total score | 81.2 ± 6.7 | 82.1 ± 6.9 | 1.1 (-1.6, 3.8) | 0.417 |

***nrRYGB:*** *Non-ring augmented roux en-Y gastric bypass,* ***rRYGB:*** *ring augmented roux en-Y* ***gastric*** *bypass* ***PHC:*** physical health composite score***, MHC:*** mental health composite score
