# Supplementary material for: Three-Year Results of Comparison Between Ring- versus Non-ring-Augmented Roux-en-Y Gastric Bypass: A Randomized Control Trial
Source: Obes Surg. 2025 Jul 17;35(8):2812–27. doi: 10.1007/s11695-025-08034-w (PMC12380956; doi:10.1007/s11695-025-08034-w)
Supplement: Supplementary file 8 — Supplementary file8 (DOCX 16 KB) [file 11695_2025_8034_MOESM8_ESM.docx]

**Appendix 8: GEE analysis for the mean difference in RAND SF-36 domains at year 3 relative to baseline adjusted for the surgery type (main effects)**

| **term** | **Mean difference (95% CI)** | ***p*** |
| --- | --- | --- |
| Physical functioning |  |  |
| After vs before | 35.4 (34.3, 36.5) | < 0.001* |
| rRYGB vs nrRYGB | 0.5 (-0.6, 1.6) | 0.333 |
| Role limitations physical |  |  |
| After vs before | 29.6 (28.4, 30.9) | < 0.001* |
| rRYGB vs nrRYGB | 0.7 (-0.5, 1.9) | 0.270 |
| Bodily pain |  |  |
| After vs before | 12.1 (10.8, 13.5) | < 0.001* |
| rRYGB vs nrRYGB | 0.5 (-0.8, 1.8) | 0.448 |
| General health perception |  |  |
| After vs before | 22.6 (21.2, 24.0) | < 0.001* |
| rRYGB vs nrRYGB | 0.3 (-1.1, 1.7) | 0.682 |
| Social functioning |  |  |
| After vs before | 20.0 (18.6, 21.4) | < 0.001* |
| rRYGB vs nrRYGB | 0.6 (-0.9, 2.0) | 0.453 |
| Role limitations emotional |  |  |
| After vs before | 30.0 (28.5, 31.6) | < 0.001* |
| rRYGB vs nrRYGB | 0.5 (-1.0, 2.0) | 0.527 |
| Energy/fatigue |  |  |
| After vs before | 18.2 (16.6, 19.9) | < 0.001* |
| rRYGB vs nrRYGB | -0.3 (-1.9, 1.4) | 0.762 |
| Emotional |  |  |
| After vs before | 21.4 (19.8, 23.1) | < 0.001* |
| rRYGB vs nrRYGB | -0.3 (-1.9, 1.4) | 0.757 |
| PHC |  |  |
| After vs before | 24.9 (23.7, 26.2) | < 0.001* |
| rRYGB vs nrRYGB | 0.5 (-0.7, 1.7) | 0.405 |
| MHC |  |  |
| After vs before | 22.4 (20.9, 23.9) | < 0.001* |
| rRYGB vs nrRYGB | 0.1 (-1.4, 1.7) | 0.861 |
| total score |  |  |
| After vs before | 23.7 (22.4, 25.0) | < 0.001* |
| rRYGB vs nrRYGB | 0.3 (-1.0, 1.7) | 0.635 |

***nrRYGB:*** *Non-ring augmented roux en-Y gastric bypass,* ***rRYGB:*** *ring augmented roux en-Y* ***gastric*** *bypass* ***PHC:*** physical health composite score***, MHC:*** mental health composite score. *Statistically significant (p < 0.05)
