# Supplementary material for: Three-Year Results of Comparison Between Ring- versus Non-ring-Augmented Roux-en-Y Gastric Bypass: A Randomized Control Trial
Source: Obes Surg. 2025 Jul 17;35(8):2812–27. doi: 10.1007/s11695-025-08034-w (PMC12380956; doi:10.1007/s11695-025-08034-w)
Supplement: Supplementary file 9 — Supplementary file9 (DOCX 15 KB) [file 11695_2025_8034_MOESM9_ESM.docx]

**Appendix 9: Changes in associated medical problems within nrRYGB and rRYGB groups at 3 years of follow-up.**

|  | **nrRYGB (n = 92)** | | | **rRYGB (n = 96)** | | |
| --- | --- | --- | --- | --- | --- | --- |
|  | Pre-operative | Post-operative  3-years | p | Pre-operative | Post-operative  3-years | p |
| Osteoarthritis | 16 (17.4) | 2 (2.2) | < 0.001* | 16 (16.7) | 1 (1.0) | < 0.001* |
| Dyslipidemia | 15 (16.3) | 4 (4.3) | 0.002* | 16 (16.7) | 2 (2.1) | < 0.001* |
| DM | 11 (12.0) | 0 (0.0) | 0.003* | 14 (14.6) | 0 (0.0) | < 0.001* |
| Hypertension | 10 (10.9) | 1 (1.1) | 0.008* | 10 (10.4) | 2 (2.1) | 0.013* |
| Sleep apnea | 8 (8.7) | 0 (0.0) | 0.013* | 12 (12.5) | 0 (0.0) | 0.002* |
| cardiac | 2 (2.2) | 1 (1.1) | 1.000 | 3 (3.1) | 1 (1.0) | 0.480 |

***nrRYGB:*** *Non-ring augmented roux en-Y gastric bypass,* ***rRYGB:*** *ring augmented roux en-Y* ***gastric*** *bypass* *Statistically significant (p < .05)
